# Spatiotemporal Movement Planning and Rapid Adaptation for Manual Interaction

**DOI:** 10.1371/journal.pone.0064982

**Published:** 2013-05-28

**Authors:** Markus Huber, Aleksandra Kupferberg, Claus Lenz, Alois Knoll, Thomas Brandt, Stefan Glasauer

**Affiliations:** 1 Center for Sensorimotor Research, Institute for Clinical Neuroscience, Ludwig-Maximilian University Munich, Munich, Germany; 2 German Center for Vertigo and Balance Disorders, Ludwig-Maximilian University Munich, Munich, Germany; 3 Robotics and Embedded Systems, Department of Informatics, Technische Universität München, Munich, Germany; 4 Chair for Clinical Neuroscience, Ludwig-Maximilian University Munich, Munich, Germany; VU University Amsterdam, The Netherlands

## Abstract

Many everyday tasks require the ability of two or more individuals to coordinate their actions with others to increase efficiency. Such an increase in efficiency can often be observed even after only very few trials. Previous work suggests that such behavioral adaptation can be explained within a probabilistic framework that integrates sensory input and prior experience. Even though higher cognitive abilities such as intention recognition have been described as probabilistic estimation depending on an internal model of the other agent, it is not clear whether much simpler daily interaction is consistent with a probabilistic framework. Here, we investigate whether the mechanisms underlying efficient coordination during manual interactions can be understood as probabilistic optimization. For this purpose we studied in several experiments a simple manual handover task concentrating on the action of the receiver. We found that the duration until the receiver reacts to the handover decreases over trials, but strongly depends on the position of the handover. We then replaced the human deliverer by different types of robots to further investigate the influence of the delivering movement on the reaction of the receiver. Durations were found to depend on movement kinematics and the robot’s joint configuration. Modeling the task was based on the assumption that the receiver’s decision to act is based on the accumulated evidence for a specific handover position. The evidence for this handover position is collected from observing the hand movement of the deliverer over time and, if appropriate, by integrating this sensory likelihood with prior expectation that is updated over trials. The close match of model simulations and experimental results shows that the efficiency of handover coordination can be explained by an adaptive probabilistic fusion of a-priori expectation and online estimation.

## Introduction

The capacity of humans to anticipate and take into account action goals of co-actors is considered as being fundamental for successful manual interaction. The ease of action coordination and remarkably fluent organization of routine joint tasks may be achieved by planning and executing actions in relation to what we anticipate from our co-actors [1], [2], [3]. By continuously monitoring the actions of our partners we can interpret them in terms of their outcomes even without any explicit verbal communication. For example, while sitting at a dinner table, we recognize the intention of another person to hand over a cup of tea by observation of the other’s hand movement, but also by contextual cues such as the state of the cup, and thus are able to initiate a reaching movement for receiving the cup well before the movement of our partner is completed.

Reaching and grasping movements have extensively been investigated in laboratory settings (for review, see [4], [5]). Recently, research has begun to look at their execution in interaction scenarios involving two or more agents [6], [7], [8]. Current research in human-robot interaction illustrates that artificially designing a cooperative handover mechanism is not as simple as it may seem [9], [10]. During interactions, humans have to response to a co-actor’s movement within an intentional context. In such experiments, the reaching and grasping movement is embedded in a continuous chain of meaningfully connected actions, which seamlessly follow one after the other. In the example above, reaching for the cup of tea is preceded by the other person taking it up and is followed by placing the cup in front of us on the table. This embedding might be fundamentally different from the separable trial structure of traditional experiments in sensorimotor control.

In the present study, we aim to elucidate several of the factors that lead to seamless and efficient dyadic human interaction, using handover of an object as a representative part of everyday joint action. The coordination of movements in such a scenario with physical interaction requires fine-tuned spatiotemporal accuracy; otherwise considerable time delays are introduced by waiting for the partner’s action or by correcting the spatiotemporal mismatch. In the case of handover, the exact position of the handover, but also an estimate of the handover time, has to be determined in order to bring the hand to the right place at approximately the right time – ready for receiving the cup of tea.

While such cooperative interaction can be triggered and guided by verbal communication, we explicitly chose to investigate the non-verbal aspects of successful handover in a natural setting. In our previous experiments [11], [12], [13], we observed that the receiving subject starts reaching for the object considerably in advance of the deliverer’s hand arriving at its final position. Thus, the question arises which factors enable subjects to successfully plan and start executing a reaching movement well ahead of being able to see its final goal. We have shown that subjects in such a simple handover scenario tend to choose a spatial position that was constant with respect to the body frame of reference [12], [13]. From this result we concluded that the spatial position of the handover was strongly influenced by previous experience or other internal factors such as implicit knowledge.

In the presented work, we investigated which additional parameters influence the decision when to move where. Therefore, we analyzed the reaction times of the receiving subject in several experimental settings.

We hypothesized that the underlying planning and decision process utilizes a combination of bottom-up visual information about the partner’s movement trajectory and prior knowledge about spatial and kinematic features of the arm movement and processes this information in a way similar to what has been proposed for other cases of cue combination and inclusion of prior information [14], [15]. In addition, we assumed that the confidence in both prior knowledge and online estimation of the handover position would depend on the type of agent, which acts as partner. To this end, we replaced the human partner with two types of robots (cf. [11]), in which we also could modify the kinematic aspects of the arm movements.

Another factor leading to high efficiency is rapid learning of context-specific aspects of the task. Indeed, in a previous handover study [11], we had observed that reaction times improved over the course of a few trials, suggesting that some aspects of the specific scenario, e.g., the rhythmic timing of repetitive actions or the exact location of the handover, have been learned. To minimize the timing aspect in the present study, we triggered each handover using randomized inter-stimulus intervals. We expected that learning and thus improvement would still occur, but less so for non-human agents.

To test our assumptions, we performed five different experiments. In all experiments we investigated the reaction times of the receiving subject during handover of six wooden cubes at randomized time intervals. In the first three experiments we manipulated the handover position to test its influence on the initiation of the reaching movement. We expected an increase in reaction times with unexpected handover positions, which deviate from the naturally adopted position [13]. In experiment 4, we explored how kinematic aspects (trajectory of the deliverer’s movement) influence the reaction time of the receiver by replacing the human deliverer by a humanoid robot moving with either a spatial minimum jerk velocity profile [16] or with a trapezoidal velocity profile in joint coordinates [17]. In experiment 5, an industrial robot with a non-human joint configuration was used in order to test the role of the deliverer's appearance on the reaction times of the receiver.

Finally, we modeled the observed processes leading to successful handover in a probabilistic framework [18], [19]. We formulated our assumptions as Bayesian estimation and decision process and performed simulations with the proposed model. A comparison of the simulated results with the experiments supports our overall hypothesis that during handover, the receiver’s ability to predict the actions of the deliverer and prior knowledge of the task are crucial for its effectiveness.

## Materials and Methods

### Participants

The experiments were approved by the ethics committee of the medical faculty of the LMU, conducted in accordance with the Declaration of Helsinki, and all participants gave their written informed consent. All subjects were right-handed university students (age 20–35, mean age was 24 (SD 3) years).

#### Experiment 1

17 pairs of subjects, consisting of a receiver and a deliverer, were tested. Data from one pair had to be excluded from the analysis due to the delayed reaction of the deliverer to the first acoustic signal indicating the start of the movement.

#### Experiment 2

20 subjects participated as receiver. Data of three subjects has been removed due to hand trajectories that strongly differed from data obtained from the rest of the subjects. The deliverer was always the same person.

#### Experiment 3

14 subjects were tested. Data of one receiver were removed from the analysis because this subject started his movement before the deliverer. As above, the deliverer was always the same person.

#### Experiment 4

12 subjects participated. Data of two subjects were removed due to technical difficulties (sensor failure) during the measurements.

#### Experiment 5

19 subjects participated. Due to sensor problems, data of three subjects were discarded in the minimum jerk velocity condition and data of two subjects were discarded from the trapezoid velocity profile condition.

### Setup and Procedure

#### Setup

**Figure 1:**
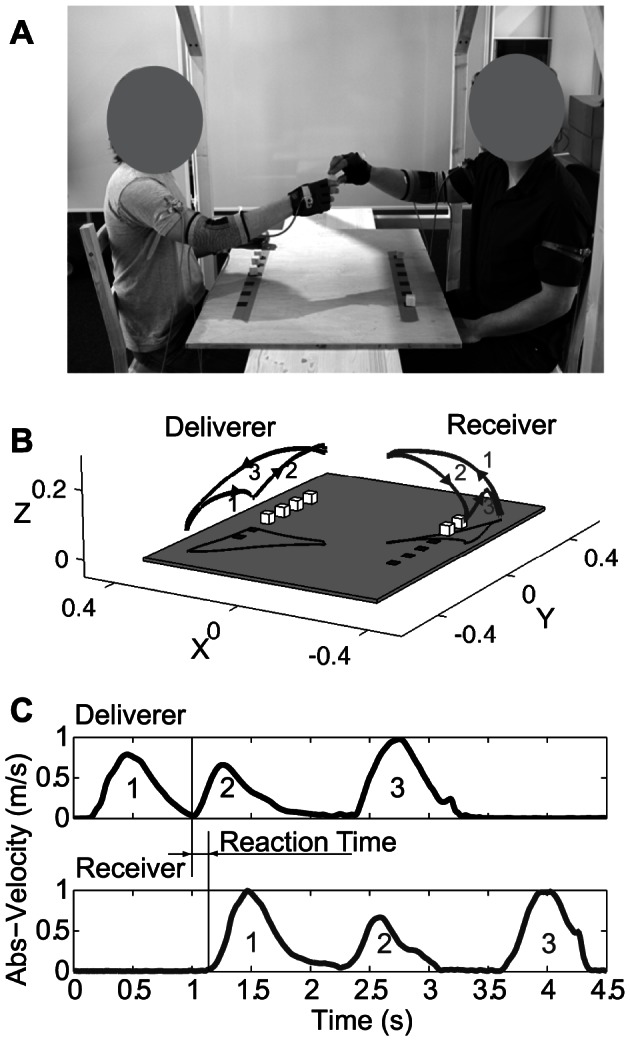
Experimental setup. A) Setup of experiment 1–3. Each handover started with the deliverer lifting the cube and passing it over to the receiver. After grasping the cube, the receiver placed it on the locations marked on the table. B) Hand trajectories in three-dimensional space for the deliverer (left, black line) and the receiver (right, grey line) for a single handover. C) The corresponding time-velocity plot shows the movement velocity of the deliverer (upper graph) and the receiver (lower graph). The reaction time was defined as time between the lifting of a cube by the deliverer and the start of the receiver’s reaching movement. The numbers in the picture correspond to the different phases of the movement marked in Fig. 1B.

Two subjects were seated opposite of each other at a 75 cm wide table. To avoid so-called end-state effects, which refers to adjustments of the orientation of the grip depended o the context of the interaction [3], [20], the items to pass were symmetrical wooden cubes (3×3×3 cm^3^). Six of those wooden cubes were placed in a row on pre-defined marks in front of the deliverer (Fig. 1A). Corresponding marks at the other side of the table served as targets for placing the cubes after each handover. The distance between the two opposite rows of marks was 50 cm. One subject served as deliverer, the other as receiver of the cubes. The receiving subject was instructed to accept each cube and place it on the marks. Both, the receiver and the deliverer were instructed to perform the task in a natural and comfortable way. No instruction about the task speed was given. The receiving subject could freely decide when to start his movement. Each subject received exactly 6 cubes, i.e., there were no repetitions and no training. The experiment was finished after the 6 handovers.

#### Procedure Experiment 1

The deliverer was required to grasp the cubes one after the other and to hand them over to the receiver. The start of each trial was indicated by an acoustical signal played to the deliverer via the headphones. The duration of the inter-trial intervals was randomized and ranged from 1s to 3s to prevent the delivering subjects from adopting a periodic pattern, which could be used by the receiver as temporal cue. The receiver was required to grasp the cubes and put them on the table near each other at the predefined marks. There was no instruction on the handover position; the deliverer could freely choose the handover position.

#### Procedure Experiment 2

**Figure 2:**
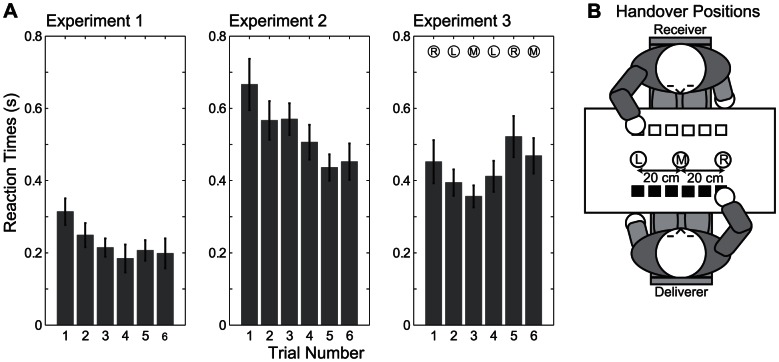
Reaction times in human-human handover tasks. A) Average reaction times for the six trials in the experiment 1–3. Error bars denote standard error of the mean. The handover position in experiment 1 was determined by the deliverer and corresponded approximately to position M in Fig. 2B. For experiment 2, the deliverer was instructed to use position R as handover position. In experiment 3, the handover position was alternated every trial according to the letters shown above the bar plot. B) Setup showing the different handover positions used the experiments. In experiment 1 the deliverers chose position M, in experiment 2 the deliverer was instructed to use position R and in experiment 3 the deliverer was instructed to use the sequence R L M L R M as handover positions.

We repeated the previous experiment with the only difference that the handover position was shifted 20 cm to the right of the deliverer (see Fig. 2B, position R). Since the deliverer had to be trained to move his hand to this unnatural handover position, the same person was used as delivering partner for all subjects. The receivers were not informed that deliverer was trained to perform handovers to an unexpected position.

#### Procedure Experiment 3

Three different handover positions were used as seen from the viewpoint of the deliverer: middle (natural handover position as described above), right (20 cm to the right) and left (20 cm to the left) (see Fig. 2B). The order of the positions during the six trials was the same for every subject: 1) right, 2) left, 3) middle, 4) left, 5) right, 6) middle. Since the deliver had to be trained to move his hand to the different unnatural handover positions, the same deliverer was used as a counterpart for all subjects. The receivers were not informed about the handover position sequence or that they were acting with a trained deliverer.

#### Procedure Experiment 4

**Figure 3:**
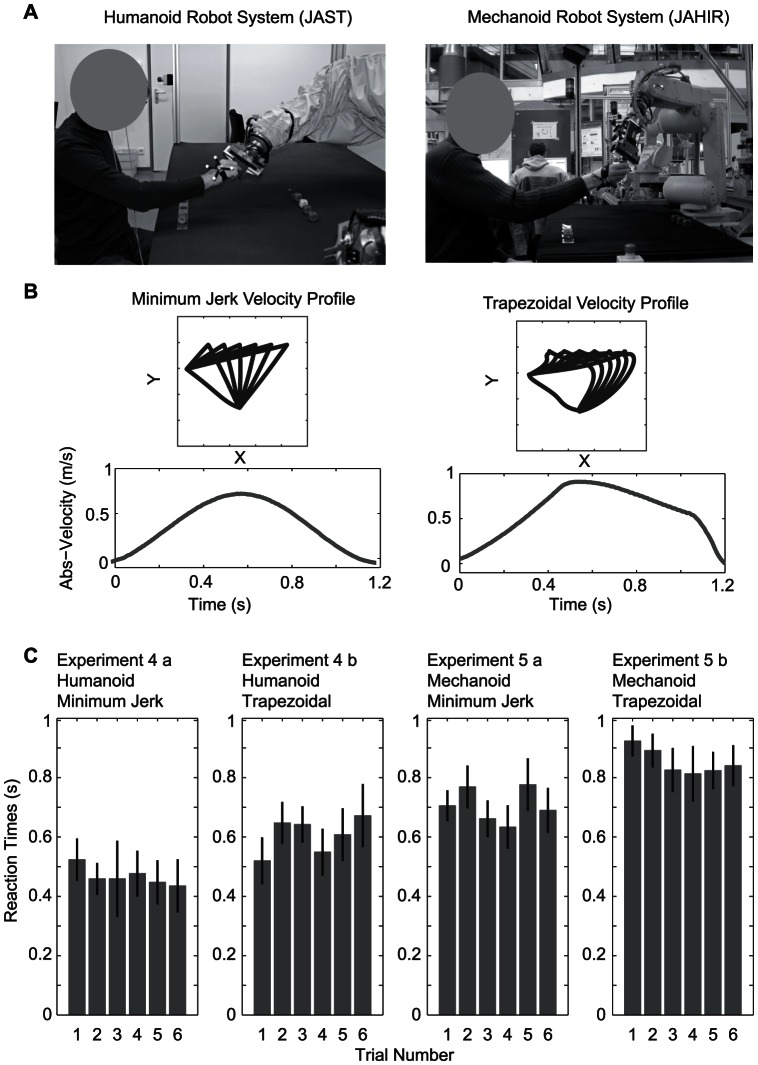
Reaction times in handover task with robot systems. A) Set up of the handover experiments with the humanoid robot system JAST used in the experiment 4 (left) and the industrial robot system JAHIR used in the experiment 5 (right). B) Trajectory (projection on XY plane) and absolute velocity plots for the minimum jerk velocity profile (left) and trapezoidal velocity profile (right). C) Reaction times during the six trials for the experiment 4 (left) and experiment 5 (right). The first plot for each experiment shows the reaction times for the minimum jerk velocity profile and the second plot for the trapezoid velocity profile.

The humanoid robot JAST [21] (see Fig. 3A left side) was programmed to replace the deliverer. The robot system had to hand over one cube after the other at a predefined handover position, which was determined as the natural handover position for the receiving subject [13]. The duration of the movement of the robotic hand (1.2 s) was approximately matched to the movement of the human deliverer determined in the first experiment (1.04 s). An exact match could not be achieved due to joint velocity limits in the robot system. The subject's task was to receive the cubes one after the other from the robot and put them on the predefined marks on the table. After each trial, the robot returned its hand to the initial starting position in the midair. The experiment consisted of two conditions. In the first condition, the robot moved with a minimum-jerk trajectory in spatial coordinates [16] (experiment 4a). In the second condition, the robot moved with a trapezoidal velocity profile in joint coordinates [17] (experiment 4b). Each subject participated in both conditions with the order of the conditions being counterbalanced between subjects.

#### Procedure Experiment 5

The setup was similar to the previous experiment with the difference that in contrast to the humanoid robot JAST, the industrial robotic system JAHIR [22] (see Fig. 3A right side) was used. JAHIR had only one arm without a torso and a head. The arm was fixed on the table at its basis and thus did not have a human-like joint configuration and appearance. Experiment 5 again consisted of two conditions: in the first condition, the robot arm moved with a spatial minimum jerk velocity profile (experiment 5a) and in the second condition with trapezoidal velocity profile in joint space (experiment 5b). The movement duration was equal to the one used in experiment 4.

### Data Acquisition

The magnetic-field-based motion tracking system Liberty™ (Polhemus) was used to record the hand movements during experiments 1–3. A small sensor (2×2×1.6 cm^3^) was fixed to the back of the deliverer's and receiver's hand to record their movement kinematics with an acquisition rate of 240 Hz. For tracking the hand of the human subject in experiment 4–5 we used the acoustic-based tracking device IS-600 (Intersense) with a sampling frequency of 20 Hz. The sensor was again placed at the back of the subjects' hands.

Fig. 1B shows an example of the trajectories of the deliverer’s (black line) and the receiver’s (grey line) hand for one handover. The corresponding time-velocity plots are shown in Fig. 1C: The first velocity peak in the deliverer's movement (Fig. 1C top) occurred while moving his hand toward the cube (1), the second while passing over the cube to the receiver (2), and the third (3) when lowering the hand to put it back on the table. The handover took place at the time point when the deliverer's movement velocity reached its plateau after the second peak. The first velocity peak of the receiver (Fig. 1C bottom) occurred when he raised his hand to grasp the cube (1), the second when he placed the cube on one of the marks on the table (2) and the third, when he returned to the starting position.

### Data Analysis

The data were analyzed in Matlab 7/8 (The Mathworks) and Statistica 6.1 (Statsoft). A Gaussian low-pass filter was used to filter the hand-position data. For data recorded with Polhemus Liberty tracking device, a cut-off frequency of 48 Hz was applied, for data from the IS-600 tracking device, 10 Hz cut-off was used. The dependent variable was the reaction time of the receiver, defined as the time delay between the start of the deliverer's movement for lifting a cube and the start of the receiver's reaching movement (see Fig. 1C). The start of the receiving movement was defined as the time point at which the velocity of the movement reached a threshold of 0.01m/s. The start of the delivering movement was defined as the time point at which the absolute velocity was 1) below 0.3 m/s and 2) reached its first minimum after grasping the cube.

The normality assumption for the reaction times was checked using the Lilliefors test. For all conditions, the normality assumption could not be rejected (all p>0.15). The reaction times were analyzed using repeated measures ANOVA. For experiments 1–3, the ANOVA had one within-subject factor *trial number* (6 levels) and one between-subject factor *experiment* (3 levels for experiments 1–3). For experiments 4-5, there where two within-subject factors *trial number* (6 levels) and *velocity profile* (2 levels) and one between-subject factor *robot* (2 levels). The Greenhouse-Geyser correction was used to correct for violations of the sphericity assumption whenever necessary (given as adj.p). Bonferroni correction was used to account for multiple testing, if required, by adjusting the p-value accordingly (given as adj.p). Further analysis of trial-to-trial changes was done using a regression approach followed by an ANOVA of the regression slopes. A p-level of p≤0.05 was considered significant.

## Results

### Experiment 1-3

We hypothesized that the receiver’s reaction in a handover task is based on the evidence for a specific handover position. This evidence can result 1) from prior expectation gained through experience either from general experience or from previous experimental trials and 2) from observing the hand movement of the deliverer. To test this hypothesis we performed three different experiments, in which two human participants performed six consecutive handovers. The experiments are organized as follows:

- In experiment 1, deliverers performed the handover at a natural position, which turned out to be close to the middle between their body positions (position M in Fig. 2B) confirming our previous study [13]. This experiment served as baseline.
- In experiment 2 we tested whether a violation of the expectation of a natural handover position would lead to increased reaction times. This experiment also tested whether subjects observe the deliverer’s trajectory in order to estimate the handover position or whether they just react to movement initiation, but then reach to the expected position without taking into account the actual movement. The handover position in this experiment was a predefined position located 20 cm to the right of the deliverer (Fig. 2B, position R).
- In experiment 3, the deliverer changed the handover position for each trial (Fig. 2B, positions L, M, or R). Thus, in this experiment, we investigated whether online adaptation seen as decreasing reaction times in experiments 1 and 2 is caused by using the preceding trial to build a new expectation of the handover position.

The resulting mean reaction times for these three experiments are shown in Fig. 2A. We analyzed the reaction times with repeated measures ANOVA with one between-subjects factor (experiment 1,2,3) and one within-subject factor (trial number 1-6). Both main effects and their interaction became significant (experiment F(2,43) = 26.1, p<0.0001; trial number F(5,215) = 3.83, adj.p = 0.005; experiment x trial: F(10,215) = 3.00, adj.p = 0.004). A post-hoc test (Bonferroni corrected) revealed that the mean reaction time was significantly smaller (both p<0.001) in experiment 1 (0.23s, SD 0.14s) than in 2 (0.53s, SD 0.22s) or 3 (0.43s, SD 0.17s). No difference in mean reaction time was found between experiments 2 and 3 (p = 0.105).

From Fig. 2A it can be seen that reaction time decreased over trials for experiments 1 and 2, but not for experiment 3. This suggests that subjects were able to improve their performance in experiments 1 and 2, but not in experiment 3. To further analyze this trial dependence of reaction times, we used linear regression with trial number as independent variable to fit a line to the individual data, resulting in one slope per subject. We then analyzed the slopes. As expected from Fig. 2A, a one-way ANOVA with experiment as factor showed that slopes differed significantly between experiments (F(2,43) = 7.5, p = 0.0016). Mean slopes were -0.0210 s/trial (SD 0.0282), –0.0435 s/trial (SD 0.0532) and 0.0148 s/trial (SD 0.0353) for the three experiments respectively. Bonferroni-corrected t-tests showed that for experiment 1 (adj.p = 0.028) and experiment 2 (adj.p = 0.012), but not experiment 3 (adj.p = 0.47), the mean slope was significantly different from zero confirming the trial-to-trial adaptation seen in Fig. 2A.

**Figure 4:**
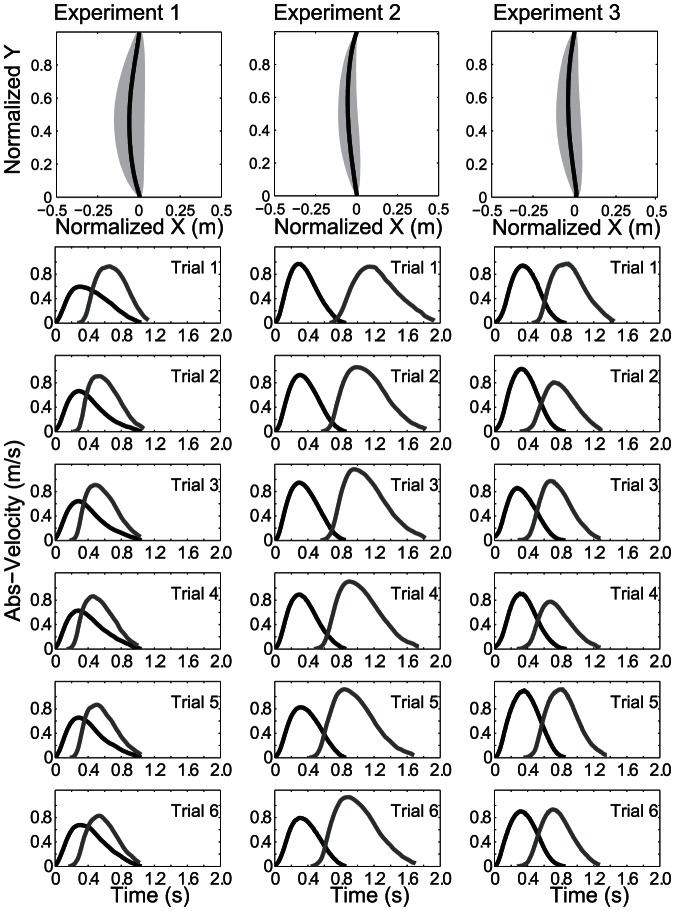
Average trajectory and velocity profiles. The first row shows the trajectories for receiver’s hand movement. The shaded area around trajectory traces represents the standard deviation of the lateral component x. Row 2–7 shows the time-velocity plot for the hand movements of the deliverer (black line) and the receiver (grey line) for the six trials of experiment 1–3.

The averaged trajectories of the receivers’ reaching movements for all experiments are displayed in Fig. 4 (first column). Note that in order to visually compare the trajectories and their variability we normalized all trajectories to a common start- and endpoint for the figure. The average movement duration of the receiving subjects was about 1 sec, as shown in Fig. 4 (first column, 2^nd^ to 7^th^ row). Note also that even in the first trials, the receiver on average started his movement before the deliverer reached the handover position (Fig. 4). The averaged trajectories are straight and the velocity profiles are bell shaped in all cases (Fig. 4). Inspection of individual trajectories and velocity profiles showed that four receiving movements (3.92%; three different subjects) in experiment 2 and three movements in experiment 3 (3.85%; three different subjects) showed properties of an online correction, such as a longer declaration phase or more than one maximum [23], [16]. This indicates that there was hardly any online correction during the receiving movement in general. The absence of online correction shows that the receivers could estimate the handover position well enough from observing the trajectory to be able to make straight movements to the correct handover position. This suggests that the receiver’s reaction is triggered by the evidence of the handover position.

The three experiments taken together show that violating the expected natural handover position leads to increased reaction times. The receiving subjects took into account the deliverer’s movement trajectory, rather than just reacting to movement onset, and recognized that the movement aimed at a new handover position. The receiving movement was initiated only when the future handover position was predicted correctly, as shown by the almost missing online correction. Experiment 3 showed that online adaptation of reaction times depends on a consistent handover position over trials.

### Experiment 4 and 5

The first experiment indicated that subjects could estimate the timing of the handover in order to reach the expected handover position approximately at the same time as the deliverer (see velocity profiles in Fig. 4, first column). We assumed that motion kinematics play a crucial role for this estimation. While the experimental modification of the position of the handover could easily be done in the realistic human-human experiment, changing kinematic aspects of the handover was not possible in such a setting. Thus, we replaced the human deliverer by two different robotic systems (Fig. 3A), which allowed us to independently manipulate the appearance of the robot and the movement kinematics of the arm motion while keeping all other aspects of the experiment constant. Varying the appearance of the delivering agent was necessary, because we expected that appearance plays a role in how much confidence subjects assigned to their prediction of the handover position. We implemented two velocity profiles in the movement of the robotic arm – a human inspired minimum jerk velocity profile [16] and a trapezoidal velocity profile in joint coordinates [17], which is widely used in industrial robots. The latter provides industries with the opportunity to use a constant maximum acceleration, which is very time efficient. The experiments consisted thus of two conditions: handing over the cubes a) using a minimum-jerk trajectory in spatial coordinates, (displayed in Fig. 3B left side) and b) using a trapezoidal velocity profile in joint coordinates (displayed in Fig. 3B right side). In experiment 4, the humanoid robotic system JAST (Joint Action Science Technology, see Fig. 3A, left side) described in [21] was used. The industrial robot system JAHIR (Joint Action for Humans and Industrial Robots, see Fig. 3A, right side) [22] was used in experiment 5. Preliminary results of experiment 4 were published in [11].

A repeated measures ANOVA of the reaction times in experiments 4 and 5 showed significant effects of robot type (humanoid or industrial) [F(1,23) = 14.21, p = 0.001] and velocity profile [F(1,23) = 15.74, p<0.001], but no effect of trial or interactions between the factors (Fig. 3C). The trapezoidal velocity profile led to an increase of reaction time for the interaction with both robotic partners. Reaction times for the industrial robot were longer than for the humanoid robot.

The average reaction times for the humanoid robot (experiment 4) were 0.47s (SD = 0.25s) for the minimum jerk profile (Fig 3C, experiment 4a) and 0.61s (SD = 0.27s) for the trapezoidal velocity profile (Fig 3C, experiment 4b). Further, reaction times were significantly longer than in experiment 1 for both the minimum jerk (Bonferroni corrected t-test, adj.p <0.005) and trapezoidal velocity profile (Bonferroni corrected t-test, adj.p <0.0005) conditions. For the industrial robots, reaction times increased to 0.71s (SD = 0.27s) for the minimum jerk velocity profile (Fig. 3C experiment 5a) and 0.85s (SD = 0.27s) for the trapezoidal velocity profile (Fig. 3C experiment 5b). Again, reaction times in both the minimum jerk and trapezoidal velocity conditions were significantly longer for the industrial robot than for the human partner as the deliverer (Bonferroni corrected t-test, both adj.p<0.0005).

These two experiments thus showed that reaction times depend on the type of agent and on movement kinematics. A movement profile similar to biological motion allowed faster reaction times than a ‘robotic’ motion profile despite having exactly the same durations and start and endpoints. Online adaptation was, on average, not present for robotic agents as deliverer despite constant handover positions over trials.

### Model

**Figure 5:**
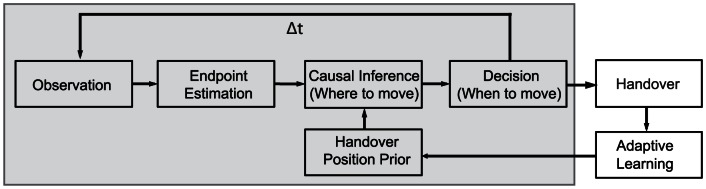
A scheme of the model. The model starts with the observation of the deliverer’s movement. The grey box comprises all components relevant within a single handover trial. The receiver is estimating the endpoint of the movement. Causal inference on the prior handover position and the endpoint estimation is used to calculate the probability of having a natural or unnatural handover. Depending on this result, the posterior distribution is calculated and used to determine the most probable handover position. The decision to act and initiate the hand movement is made if the handover position is determined with a sufficient level of evidence. After the handover the prior is updated by an adaptive learning rule.

**Figure 6:**
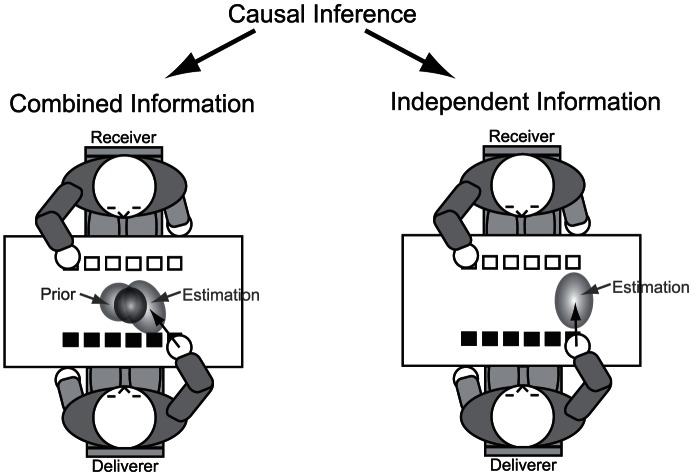
Causal Inference model. In the handover experiments the receiver has to determine the handover position before initiating his hand movement. For this purpose the receiver has to decide whether he combines the prior and the endpoint estimation of the deliverer’s movement (left side), or whether he neglects information on the prior handover position (right side). Causal inference solves this problem by comparing the probabilities of the two cases and deriving optimal predictions from this.

The stereotypic behavior of the subjects in the different experiments allows us to infer the basic mechanisms of the handover tasks. We developed a probabilistic formulation (framework, see Fig. 5: model’s principle structure) that is based on the assumption that the receiver reacts as soon as the handover position can be determined with a sufficient degree of confidence (Fig. 5 “Decision”). This requires predicting the handover position as well as the reliability of the prediction (Fig. 5 “Causal Inference”). For this purpose the receiver has two sources of information: the predicted handover position based on the observed movement (hereafter called the “endpoint estimation”) and the assumed prior about the handover position. If the endpoint estimation is compatible with the prior, the receiving subject integrates the information to get a fast and accurate prediction. If the endpoint estimation conflicts with the prior expectation, the current prior is rejected and changed to a uniform distribution. Cues for the endpoint estimation could be, e.g., the velocity profile and trajectory of the delivering movement, which can be compared to familiar trajectories to infer the possible endpoint. To estimate the handover position and its evidence a decision has to be made, whether both sources of information or just the online estimation of the movement endpoint are taken into account. This decision can be reached through a Bayesian inference process (see Fig. 6) similar to causal inference [24] in multimodal perception.

In the following we define the integration of the handover position prior and the endpoint estimation as the natural handover case *C = N*, while an unexpected handover position is defined as the unnatural case *C = UN*. In our framework, an ideal observer is facing the problem of inferring whether the endpoint estimation and the prior should be integrated or if the prior should be ignored. This inference is performed by means of statistical decision theory [25]. Using Bayes’ rule we can calculate the probabilities of a natural (*C = N*) or an unnatural (*C = UN*) handover for a given handover position. These two probabilities can simply be compared if we assume that the two possible misclassification cases are treated equally.

Applying Bayes’ rule to calculate the probability of a natural or an unnatural handover occurrence, leads to:
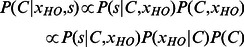
(1)where 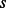 represents the estimated handover position based on sensory data, and 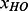
is the hypothesized handover position. 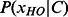 represents the prior belief about where a handover will take place given a natural handover. 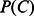 represents the prior of the handover case and can be interpreted as the trust of the receiver that a natural (*C = N*) or unnatural (*C = UN*) handover will occur. In this way, the inference whether a handover is natural or not is now formulated as a combination of the endpoint estimation 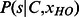 and the handover position prior 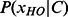. Since the endpoint estimation is assumed to be independent from the handover case, the formula for the endpoint estimation reduces to:
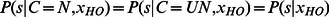
(2)


While all factors are assumed to be a Gaussian distribution, the handover position prior for the unnatural case 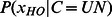 it is set to a uniform distribution. With our model assumptions Equation 1 can now be formulated as:
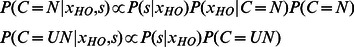
(3)


The posterior in equation 3 depends now on whether or not the handover position prior improves the prediction from the endpoint estimation.

Since the posterior probabilities 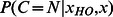 and 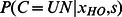 add to 1, we assume that the model reports a natural handover when: 
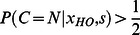
(4)


For more details about the “causal inference” process please refer to Text S1. Thus, the estimation of a normal handover amounts to a Bayesian model selection problem. It is mathematically similar to a Gaussian mixture model proposed for depth perception [26].

Since we now know via Equation 4 whether there is a natural or unnatural handover case, we can estimate the optimal handover position. Since we assume all distributions to be Gaussians, we can derive an analytic solution for the most likely handover position by minimizing the mean squared error, which here is consistent with the maximum-a-posteriori solution [24]. If the model reports a natural handover case *C* = *N* the most likely handover position can be calculated by:
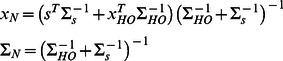
(5)where 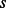 and 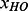 are the expectation values for the endpoint estimation and the handover position prior and 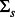 and 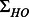 are the corresponding covariance matrices.

In case the model reports an unnatural handover *C* = *UN*, the most likely handover position reduces to:
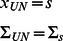
(6)because the prior handover position is set to be uniformly distributed (see Equation 3).

Using this formulation, we can infer the handover case and the handover position, given a prior handover position and endpoint estimation (Fig. 5 “Causal Inference”).

The inference of having a natural or unnatural handover (Equation 4) is continuously updated, because the uncertainty of the endpoint estimation and its position represented by 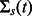 and 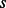 are time dependent. This is due to the fact that longer observation of the deliverer’s movements will increase the evidence for the estimated endpoint. Therefore, the covariance matrix for the endpoint estimation depends on the observation. Fig. S1 in Text S1 shows a simulated development of the diagonal elements of the covariance matrix 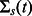. During a handover, we assume the covariance matrix of the handover position prior and the mean of the prior to be constant (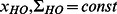). Note that in the current model implementation the estimate is updated only until the movement is initiated. To simulate occasional online corrections, the updating could be continued until reaching the handover position.

**Figure 7:**
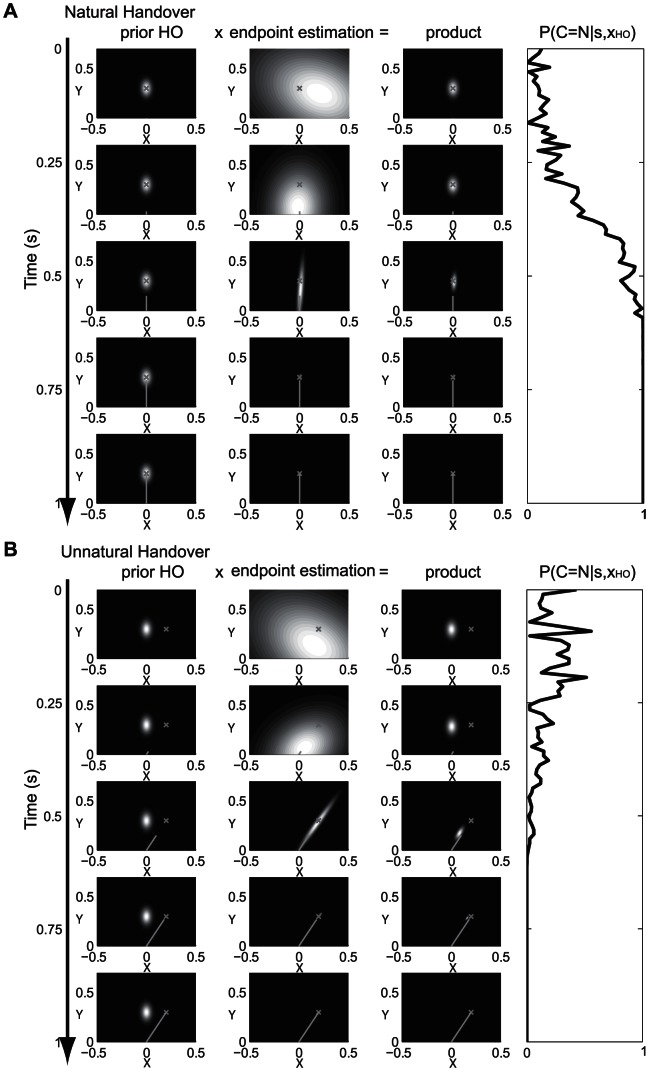
Development of the probability distributions during a handover. A) Natural handover case: The figure displays the probability distributions of the prior handover position (first column), the estimated endpoint of the deliverer’s movement (second column) and the product of the prior and estimate (third column) in spatial coordinates. The x is the real handover position; the line towards the x indicates the part of the deliverer’s handover trajectory, which at this time point is observed from the receiver. The rows in the figure display the development in time during one handover action. The time development of the probability *P(C = N|s,x_HO_)* that there will be a natural handover is shown in the column on the right side. During the handover, the prior probability distribution (first column) stays constant. At the beginning it is not possible to accurately estimate the movement endpoint, which results in a very large uncertainty. While observing more of the trajectory the estimated probability distribution becomes sharper and more accurate. If the prior and estimated distribution lie close together *P(C = N|s,x_HO_)* converges to 1. B) Unnatural handover case: In this case the prior probability distribution does not match the real handover position (first column). A longer observation allows a more accurate estimate of the movement endpoint (second column). The product of both probability distributions leads to a discrepancy of the estimated handover position (maximum of the probability distribution in column 3) and the real handover position (marked as x). Because of the difference in the estimated handover position and the prior distributions the posterior probability *P(C = N|s,x_HO_)* converges to 0.

Fig. 7 displays the spatial development of the probability distributions for prior and estimated endpoint, as well as the product of the two distributions over the time of one handover action. Fig. 7A shows the probability distributions for the case where a handover takes place at a natural position. The probability of having a natural handover 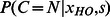 is displayed in the right column of Fig. 7. If the prior and estimated distributions lies close to each other the probability of having a natural handover increases. Fig. 7B shows the probability distributions for an unnatural handover position, in which the prior distribution does not correspond to the estimated endpoint position. In this case the product of the probability distribution leads to an inaccurate description of the handover position. Thus, a natural handover becomes unlikely over the course of time.

Up to now, it is still unclear at which point in time the receiver should decide to initiate his own movement. A simple decision rule is to apply a criterion to the decision process [27]. In our case it is reasonable for the receiver to react when the handover position is determined to a sufficient degree of confidence. If this is not the case, the receiver has to continue observing the deliverer’s movement in order to increase the evidence (Fig. 5 “Decision”). Therefore, we introduce a decision value (DV) in the presented model whose magnitude reflects this confidence. The decision value is calculated as the mean of the variances along the eigenvectors of the covariance matrix of the most likely handover position. Which covariance matrix (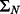 or 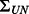) has to be used depends on the handover case reported by the causal inference process (Equation 4). Equation 7 describes the DV, where 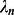 is the nth eigenvalue and variance of the covariance matrix (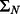 or 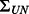). The decision value reflects the level of evidence of the handover position or how sharply the handover position is determined. In the following we will call the DV the “averaged variance” of the handover position:
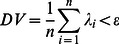
(7)


The decision criterion is that the receiver will react when the decision value falls below a threshold 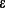. For the natural handover case (*N*), the integration of the prior and endpoint estimation leads to a faster decrease of the decision value than in an unnatural case, where only the endpoint estimation delivers relevant information (*UN*). Therefore, the criterion is reached faster than in the unnatural case, allowing a faster initiation of the reaching movement. The initiated movement is planned to reach the most likely handover position 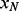 or 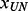, dependent on the determined handover case.

So far the model is able to explain the general difference in reaction times for natural and unnatural handover experiments. The model does not yet explain the decrease of the reaction times over the consecutive handover trials in experiment 1 and 2. The adaptation in experiment 1 and 2 can be explained by learning effects similar to [15], [28], [29] in which the learning or update of priors is investigated. The experimentally observed rapid decrease of reaction time indicates a rapid update of the prior’s mean. A fast learning rate for the prior’s mean and much slower update of the variance has also been observed in other experiments [28]. Therefore, to update the prior after each handover we implemented a two-dimensional version of a previously proposed adaptive Bayesian model [29] with different learning rates for the prior’s mean and covariance matrix:
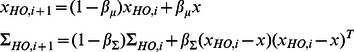
(8)where i denotes the i-th handover and 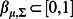 are the update rates for the prior’s mean 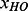 and covariance matrix 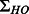. 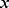 represents the real handover position. In the scheme of our model (Fig. 5) this takes place in “Adaptive Learning”.

### Model Validation

To validate the model’s predictions about the reaction times of the receiver, we integrated the model in a simulation framework. The simulations were performed using the same handover parameters as in the experiments. The simulation used two planar dimensions to reduce the complexity. Noisy minimum jerk trajectories with endpoints corresponding to the experimental condition and duration of 1 sec were generated and fed into the framework. For the robot experiment the duration of the trajectories was adjusted to 1.2 sec to match the experiments. For the robot experiments 4b and 5b, two-dimensional projections of the experimental robot trajectories were used.

The model was able to describe the average behavior seen in our experiments. The implementation of the model for the simulation required several assumptions. Since we do not know exactly which cues humans observe when estimating the endpoint of movements, the implementation of endpoint estimation in the simulation is a simplified assumption of how this might be achieved in humans.

For the simulation we needed to adjust several parameters of the model to match the size of the reaction times and adaptation:

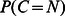
: the prior probability of having a natural handover,

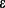
: the threshold of the decision value,

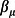
,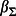: the learning rates for the mean and covariance of the prior distribution of the handover position.

The mean and the covariance matrix for the natural-handover prior were calculated from handover position distributions collected in [13]. All parameters where adjusted at the beginning of the simulation and stayed constant in all experiments. Details about the implementation of the model and the values of the parameters are given in Text S1.

**Figure 8:**
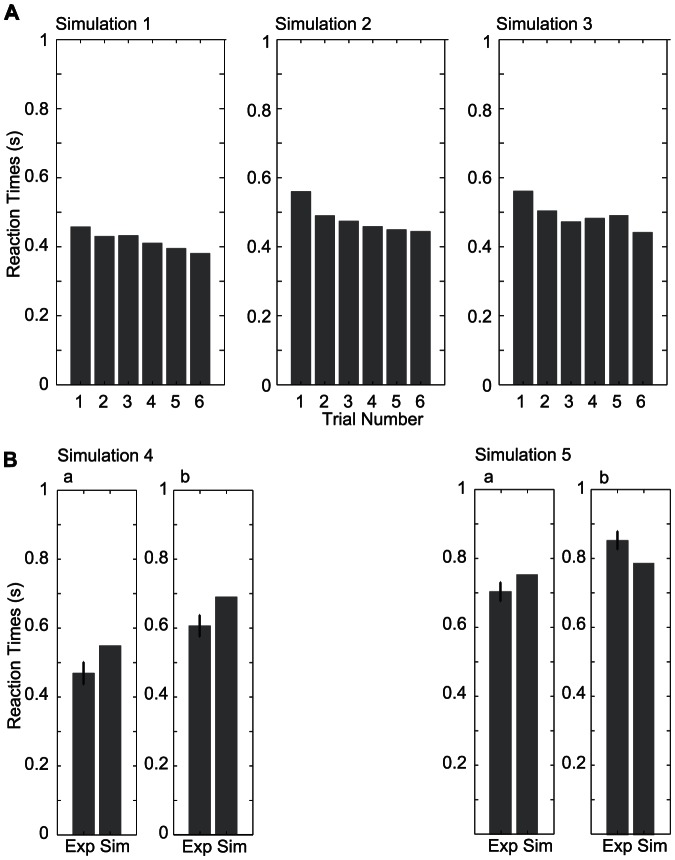
Simulated reaction times for the handover experiments. A) Simulation of the human-human experiments. Simulated reaction times within the framework outlined in Fig. 5 shows similar behaviors as the reaction times in the human-human handover experiments. Simulation 1 with natural handovers shows the relatively short reaction times compared to the other experiments and the decrease of the reaction times over the trials. Simulation 2 shows reaction times with handovers to a position 20 cm to the right. This leads to longer reaction times compared to natural handover reaction times in general but also shows a decrease of the reaction times over trials. Simulation 3 with handovers at random positions shows also longer reaction times compared to Simulation 1 but no decrease over trials. The simulation results are qualitatively consistent with the experimental measurements. B) Simulation of the robot-human experiments. The averaged simulation reaction times of the robot experiments are compared with the averaged reaction times from the experiments. In simulation 5 the threshold of the decision value is decreased, i.e., the prediction must be more precise for the receiver to initiate his movement. This leads to longer reaction times in general. The simulations with trajectories generated by trapezoidal velocity profiles (Simulation 4b and 5b) leads to increased reaction times because the estimated endpoint is not accurate for a longer observation period.

The simulation successfully captured the differences in reaction times between the experiments as well as the tendency to adapt. Fig. 8 recapitulates the simulated reaction times for all experiments.

Experiment 1 showed the shortest reactions times of all experiments together with a decrease in reaction time over trials. The simulation using the same handover positions as in experiment 1 was able to reproduce short reaction times with a decrease over trials (Fig. 8A left). The integration of prior handover position and estimated movement endpoint led to a more precise determination of the most probable handover position and thus the decision value threshold was reached fast. Because of the very small error between the handover-position prior and the actual handover position, there was a decrease of the covariance matrix of the handover position prior. Thus, in subsequent handovers the threshold of the decision value was reached faster.

The simulation of experiment 2 (handovers displaced 20 cm to the right) resulted in longer reaction times than for experiment 1 (Fig. 8A middle). The first handover was recognized to occur at an unnatural position so that a uniform prior was used. Thus, the most likely handover position was determined only through endpoint estimation and remained uncertain for a longer observation period. Therefore, the threshold of the decision value was reached much slower, which led to a longer reaction time than for the first handover trial in simulation 1. Because of the large error between the handover-position prior and the actual handover position, the covariance matrix of the handover-position prior increased and the mean drifted towards the handover position. Due to the updated prior the following handovers to the same positions are classified as compatible with the new prior, which then leads to a decrease of the reaction times over the trials.

A simulation of experiment 3 with randomized handover positions leads to an increase in reaction time compared to simulation 1 (Fig. 8A right). The simulated reaction times show interesting similarities to the measured reaction times. Like in simulation 2 the first handover was classified as an unnatural handover, due to the mismatch of the handover position prior and the estimated movement endpoint. The large error between the handover-position prior and the actual handover position led to an increase of the handover-position prior’s covariance matrix and a shift of its mean. The updated covariance matrix was large enough to lead to classification of the subsequent handover to a new position with a distance of 40 cm to the previous one as ‘natural’ handover. Thus, the prior is used to determine the handover position, which leads to decrease in reaction time for the second handover. All subsequent handovers are also classified as natural handovers. Therefore, update of the handover-position prior’s mean and covariance matrix according to the different handover positions determines the length of the simulated reaction times: a large error between the current handover position and the prior leads to a large covariance matrix and thus to longer reaction time for the subsequent handover.

In the robot experiments, where the handover position was similar to experiment 1, we found longer reaction times for the humanoid robot using minimum jerk trajectories than for the human-human experiments. This was captured by the simulations without the need to change parameters, because it was a direct consequence of the longer duration of the robot trajectories (1.2 s) as compared to the human trajectories (approx. 1 sec). For an accurate estimation of movement endpoint, the time for observing a movement with longer duration has to be extended, because its kinematic features, e.g. the acceleration, are not as distinct as for faster movements. Furthermore, different reaction times were found between a robot system using a minimum-jerk and an industrial velocity profile (see Fig. 8). Interestingly, the model also predicts different reaction times, because curved trajectories, such as the trajectories generated by the trapezoidal velocity profile, lead initially to wrong endpoint estimates. Therefore the model predicts an unnatural handover for a longer observation period, even if the curved trajectory is ending in a natural handover position. This results in longer reaction times for the trapezoidal velocity profile (Fig. 8B, simulations 4b,5b). In contrast, a minimum-jerk trajectory allows a correct estimation of the movement endpoint, which results in faster classification for handover to a natural position and thus a faster reaction. However, although our model predicts an adaptation for robot experiments, we did on average not find statistically significant adaptation in the empirical data. In the model the adaption can be turned off by setting the learning rates for the prior of the handover position to 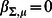. Furthermore, the effect on the robot type (longer reaction times for the industrial robot system) is not captured by the model, but can be simulated by adjusting the decision value threshold to a lower level, i.e., requiring higher accuracy, for the industrial robot system (cf. Fig. 8B, simulation 5a,b).

## Discussion

The present investigation of manual interaction demonstrates that the mechanisms underlying handover coordination between human subjects can be understood as an adaptive probabilistic optimization process. We demonstrated that in human-human handovers at a natural handover position, the receiver’s reaction times decreased systematically over the course of six trials (see Fig. 2A experiment 1). This decrease also holds if the position of the handover was unexpected in the first trial, but stayed constant over the 5 consecutive trials (see Fig. 2A experiment 2). However, when the handover position changed in every trial, no adaptation of reaction times could be observed (see Fig. 2A experiment 3).

We explain these results with a Bayesian observer model, in which two sources of information are available: a prediction about the handover position, which is estimated over time from observing the other’s hand movement and a-priori experience about the handover position. Furthermore, we assume that a causal inference process is used to decide whether to fuse this information optimally or to reject the prior and use only the estimated endpoint of the receiver's movement. The decision to move depends on the reliability of the estimate. The decrease in reaction time is explained by rapid adaptation of the handover prior.

To further investigate the influence of the endpoint estimation on the reaction time of subjects, we replaced the human deliverer by robot systems (see Fig. 3A). By using robot systems, we were able to measure the reaction times for different movements of the deliverer. We found that the reaction times depend on the type of robot and on the used velocity profile but not on the trials. For both robots, reaction times of receivers were longer if the robots moved with an artificial trapezoidal velocity profile versus a quasi-biological minimum jerk velocity profile. This can be explained by an improved capability to predict the movement endpoint when the handover movement is performed in a familiar manner in respect to appearance and movement. The suggested principles were formulated in a probabilistic framework, which combines causal inference [24], decision [27] and adaptation processes [28], [29]. Simulations of the experiments could successfully capture the key features of the experimental results.

The comparison of our findings in the human and robot experiments indicates that certain aspects of human interaction behavior are highly dependent on our expectations regarding the co-actor. Hence, our work supports the claim that human interaction behavior can hardly be understood only from experiments involving a single human participant, but that the human partner is essential to uncover the mechanisms underlying human joint action [30].

### The importance of movement prediction

Many behavioral tasks have time constrains, such as catching a ball in a sports game or turning the steering wheel while driving a car. Being successful in these tasks requires generating predictions about the environment, based on sensory information. While a long observation of the environment reduces the uncertainty of the prediction, it also increases the motor variance, because less time is left for the action. Humans are therefore assumed to trade-off sensory and movement uncertainties in a statistically optimal way [31].

During a dyadic interaction, the capacity to quickly register the intention of a teammate and react to it before his/her action sequence is completed is essential for a fluent team performance [32]. Therefore, to dynamically adapt their movements to one another, the co-workers need to take their partner’s movements into account. It has been shown, that the mere knowledge about the upcoming movement of the partner is sufficient to excite one's own motor system, because it is more advantageous to anticipate rather than react to the other's actions [33]. While watching the actions of others, even human eye movements are predictive rather than reactive [34]. Accordingly, humans often can predict how a movement will end just from watching its initiation. For instance, it has been reported that basketball players are able to determine whether another player is about to throw the ball or just mimic a throw [35], [36]. This predictive capacity may also be the reason for our ability to perform during interaction scenarios different movements simultaneously instead of successively [12].

In our experiments the receiver always reacted before the deliverer reached the end position of his movement (see Fig. 4). This indicates that action planning integrates predictions about the actions of the interacting partner. Such prediction of the handover position is necessarily based upon observing the initial kinematics of the deliverer, such as velocity and position of his hand. Despite ample evidence for the human ability to predict the outcomes of other people’s movements [35], [37] and several computational theories of how this could be achieved (e.g., [38], [39]), detailed models of how such theoretical mechanisms may operate together to yield motor action for concrete examples involving action observation are rare (for an exception see [40]). In our probabilistic framework we used the observation of the noisy hand trajectory to estimate the movement endpoint. It is likely that humans also use additional cues as predictors for the movement endpoint, e.g., observation of the multi joint movements of the arm and body or of predictive gaze shifts [34]. However, not only the estimation of the movement endpoint, but also the reliability of this estimation is important [41]. This reliability strongly depends on the amount of time that is available for the observation of the movement. A longer observation time leads to a more reliable estimate. In our simulation we solved the problem of time-dependent estimation reliability by a lookup table built from Monte-Carlo simulations (for details see Text S1), which assigns reliability depending on the proportion of the observed trajectory. While it is not clear how the brain solves the problem of time-dependent reliability, it could in principle work as in the model. Accordingly, the reliability would be learned from experience and be assigned depending on the current estimate of trajectory length.

### How prior knowledge influences the effectiveness of the handover

Several studies have convincingly demonstrated that humans try to combine information from different cues in an optimal way to minimize the uncertainty of their predictions and plan their actions accordingly [18], [14], [42]. We suppose that the actions of the receiver in our experiments are also based on such a strategy. The receiver in our experiments uses the combination of two cues for estimating the most likely handover position. The first is a prior for the handover position, which is acquired, for example, during everyday interaction. The second source of information is the estimated movement endpoint, derived from the perceived movement kinematics of the deliverer. The combination of two cues from different modalities leads to faster and more accurate reactions than a cue in only one modality, e.g. as in case of saccades when visual and auditory stimuli are jointly presented as a target [43].

Our study has shown that the receivers’ reaction times in the first experiment are the fastest compared to all other experiments. We suppose that in the first experiment, where the handover always occurs in the middle between the subjects, the expected (natural) handover-position (represented by the prior distribution) and the endpoint estimation from observing the deliverer’s movement are integrated. The reliability of the estimated handover position thus becomes a function of the reliability of the prior and of the observation time. The combination of the two cues allows the receiver to estimate the most likely handover position with a sufficient degree of confidence within a relatively small observation period, thus allowing fast reactions (see Fig. 7A).

However, combining cues does not always make sense. Two cues may either have a common cause, in which they should be integrated, or have different, independent causes, in which case they should be processed separately [44]. In the present case, the prior distribution may be appropriate, if the handover is planned at a natural position, but should be rejected, if, for some reason, the deliverer’s movement aims at a completely different location. The significantly longer reaction time for displaced handover positions in the first trial of the second experiment or in the third experiment suggested that the prior about the handover position and the estimated endpoint of the movement were treated separately. A combination of this prior and estimation would have led to an incorrect prediction because of the big discrepancy between the prior and the estimated endpoint (see Fig. 7B). We suggest that a statistical decision process, similar to causal inference [24], infers whether the current action aims to a position consistent with the prior, or if it is directed to an unexpected position. In the latter case the prior is rejected and the estimated endpoint of the receiver's movement is used. Hence a longer observation period is required to achieve a sufficient degree of confidence to elicit an action. The causal inference process in our model shows similarities to the responsibility estimation in the MOSAIC model [38], [45] except that in our case only a single forward model is applied and the selection determines whether prior experience is applicable (handover position prior) or not (uniform prior).

It has been shown that the prior expectation frequently is acquired over the course of experiments [15], [28], [29]. An update of prior expectation has also been proposed to explain why reaction times to the visual part of an audiovisual stimuli decreases, if previous audio and visual stimuli were congruent [46]. In our experiments 1 and 2, where the handover took always place at the same position, we observed a decrease of reaction times over trials. In contrast reaction times remained high when the handover positions where randomized (experiment 3). To explain this finding, we suggest that the receiver updates the handover-position prior by taking into account the error between the current handover-position prior and the real handover position. A small error leads to a small shift of the prior and to a decrease of its uncertainty, whereas a large error leads to a large shift towards the real handover position and an increase of its uncertainty. Consequently, successive consistent handover positions will lead to a decrease in reaction time as found experimentally.

In the first experiment the handovers occurred close to the prior handover position. Therefore the error was small even for the first trial. In subsequent handovers this resulted in a more accurate prior. In the second experiment the handover position was always displaced and the large difference between actual handover position and prior led to longer reaction times and an increased variance. In the subsequent trials the deviation decreased, since the handover position did not change. This allowed the prior to become more accurate and be combined with the estimated handover position again. Similar to experiment 1, this led to a faster estimation of the handover position with a sufficient degree of confidence, and therefore to shorter reaction times. In the third experiment, the changing handover positions led to large errors between the prior and the real handover positions. The large error increased the uncertainty about the prior, shifted the prior towards the last handover position, and precluded the fusion of prior and the estimated handover position. Therefore the lacking adaptation in experiment 3 can be explained as a result of the errors between the prior and the real handover position and the attempt to compensate for this error by readjusting the prior.

In cognitive science a “shared task representation” has been suggested as basis for joint action tasks. Such a representation can be used to predict the needs of the co-actor [2]. Similar to our findings in the handover experiments, a “shared task representation” might also contain several prior expectations concerning the co-actor, which are updated during the joint action. Therefore, extending the present modeling framework to include the actions of both participants could build the basis for modeling more complex joint action tasks.

### How a sufficient level of evidence triggers the reaction

In interaction tasks people have to face the problem of inferring the action of a co-actor based on uncertain observations, e.g. when inferring the co-actor’s goals [40]. But observations change over time and influence the decision when to make an action. We hypothesize that the receiver in a handover tasks reacts after acquiring sufficient evidence about the handover position. Such a strategy would allow the receiver to react with a movement straight towards the expected handover position without corrections during the movement. The experimental results support our hypothesis as we found only in very few cases evidence for online correction in the recorded arm movement trajectories and velocity profiles. Typical features of trajectories, which have been corrected online, are increased movement duration, lower peak velocity, and a longer deceleration phase [23]. Furthermore, the bell-shaped velocity profile is no longer preserved. Instead of a single global velocity maximum, there can be two or more local maxima in the velocity profile [16]. Such features were generally not observed in our experiments (see Results, Fig. 4, and Fig. S2 in Text S1 for an exception).

An alternative strategy, where the receiver starts his movement as fast as possible towards the prior handover position would require online correction of the movement to reach unexpected handover positions [47]. This in turn would increase the movement duration and length, hence decreasing the efficiency and fluency in an interaction. Furthermore, during an interaction such online corrections could be confusing for the counterpart. Thus, our experimental results indicate that the strategy in interaction scenarios is optimized towards efficiency and fluency.

In our model, we use an empirically derived fixed criterion on a value related to the confidence for the most likely handover position. The exact formulation of the value related to the evidence for the handover position is not critical for the model’s principle behavior. We chose the ‘averaged variance’ (equation 7), i.e. the ‘total variance’ divided by the number of dimensions, of the handover position distribution. This choice is supported by the close match of simulations and experimental results. Alternatively, the determinant of the covariance matrix of the handover position distribution could be used, which is called ‘generalized variance’, and which is the product of the eigenvalues.

A possible alternative for the decision to move would be a “race model” [27], [48]. For example, such a race model could implement two decision values, one based on the estimated handover position using prior experience, the other one without (uniform prior). However, since the variance (averaged or generalized) of a fused estimate is always smaller than that using the uniform prior, such a model would favor the integration of handover position prior and endpoint estimation in all handover cases, even when the endpoint estimation and the handover position prior do not match. Therefore, this mechanism would predict short reaction times and online corrections for handover to unexpected positions in contrast to our experimental results.

### Effect of movement kinematics, appearance and joint configuration on the effectiveness of handover

We hypothesized that short reaction times are mainly a result of the ability to estimate the partner’s actions both temporally and spatially. To test how this ability depends on biological movements and agent appearance we implemented two different velocity profiles, a typical robotic motion profile (trapezoidal joint velocity)[17] and a human-like minimum-jerk profile [16], in two different robotic systems: a mechanoid (JAHIR)[22] and a humanoid robot (JAST)[21], which replaced the deliverer. The experiments showed that reaction times were significantly longer for handovers with the trapezoidal velocity profile than with the minimum jerk velocity profile. Furthermore, no adaption in the reaction times could be observed suggesting that subjects were not updating their handover-position prior. These effects are observed in both robot systems. The trapezoidal velocity profile with its sudden transition from zero to full speed and vice versa makes it difficult to estimate the endpoint and duration of the movement from partial observation. Our results are consistent with previous studies on motor interference showing that observation of a humanoid robot performing incongruent movements based on prerecording of a human experimenter has a stronger influence on the own movement (higher variance on the movement trajectory) than watching the same robot move with a constant-velocity profile [49], [50]. Further studies have shown that observation of biological, but not non-biological dot motion caused motor interference [51], [52]. While the differences in reaction times according to the velocity profiles is consistent with the predictions of the model, the question why there is no adaption over trials is unclear. We suppose that the subjects’ lack of experience with robot system might have prevented a successful update of the handover position prior and thus adaptation.

In the present study, the presence of humanoid features in the robotic deliverer had a positive effect on prediction of its movement, since reaction times were shorter for the humanoid robot (JAST) [21] than for the industrial robot (JAHIR) [22] independently of the velocity profile. However, we assume that it is not so much the appearance but rather similar motility that accelerated interaction. This assumption is supported by our recent study on motor interference using the same robots [53]. The necessity of the morphological similarity of observed agent for easy prediction of its actions might be caused by the "simulation theory". This theory states that individuals recognize each other's intentions and predict each other's actions by imagining themselves in the other's position, and simulating mental states (beliefs, desires, intentions) that they would possess if they were in the other's 'shoes' [54], [55]. This procedure reduces the possible range of actors, whose intentions the observer might be able to simulate, since for simulation the observed actor should have the same motor constraints and morphological features as the observer (the "like me" hypothesis [56], [53]). Since this assumption is to some extent true in a humanoid robot but not an industrial one, the simulation procedure might be effective for attributing goals to motor actions of JAST, but not of JAHIR. Evidence supporting this assumption is also presented in a recent study using agents with a different degree of human likeness (computer, functional robot, humanoid robot, and human). The study has shown that the more anthropomorphic a machine looks like, the more the user will expect it to behave like a human and ascribe mental states to it [57].

## Conclusion

The present investigation of dyadic interaction is a first attempt to better understand the mechanisms of coordinating sequences of actions between human subjects. Based on the results of all five handover experiments, we suggest that the receiver’s behavior in handover tasks can be explained by different interacting mechanisms. From observation of the movement kinematics during an attempted handover the endpoint of the movement is estimated. The estimated endpoint becomes more precise with the increasing duration of the observation, i.e., evidence for a specific handover position is accumulated. This estimated endpoint is combined optimally with a prior for the handover position, which is acquired during everyday interaction. Comparable to causal inference in multimodal perception, estimate and prior are either fused or the prior is rejected depending on the difference between both. A fusion of prior handover position and estimated endpoint results in a more precise prediction of the handover position. The reaction time of the receiver depends on the evidence about the predicted handover position. Thus, a fusion of prior handover position and estimated endpoint results in shorter reaction times. The handover-position prior is iteratively updated after each handover, which allows adaption to the deliverer.

The similarity of simulation results and experimental data suggests that simple interaction behavior such as handover can be well explained by Bayesian estimation and decision processes combining prior knowledge and sensory input. Our work also shows that the coordination of actions between humans needs to be investigated in experiments involving human-human interaction to understand its underlying principles. In other words, even though the use of a robotic system as interaction partner can be helpful experimentally (see our experiments 4 and 5), it cannot replace investigations with a human co-actor.

## Supporting Information

Text S1 Detailed description of the model implementation and example of a movement with online correction.
(DOC)
