# Supplementary material for: Spatiotemporal Movement Planning and Rapid Adaptation for Manual Interaction
Source: PLoS One. 2013 May 28;8(5):e64982. doi: 10.1371/journal.pone.0064982 (PMC3665711; doi:10.1371/journal.pone.0064982)
Supplement: Text S1 — Detailed description of the model implementation and example of a movement with online correction. (DOC) [file pone.0064982.s001.doc]

Text S1

Supplementary text for

**Spatiotemporal movement planning and rapid adaptation for manual interaction**

Markus Huber, Aleksandra Kupferberg, Claus Lenz, Alois Knoll, Thomas Brandt, Stefan Glasauer

**Causal inference:**

Applying Bayes’ rule in order to calculate the probability distribution of a natural or an unnatural handover occurrence leads to:

$P(C|x_{HO},s)=\frac{P(s|C,x_{HO})P(C,x_{HO})}{P(s)}=\frac{P(s|C,x_{HO})P(x_{HO}|C)P(C)}{P(s)}$ (1)

where $P(s)$is a normalization factor. $s$ represents the estimated handover positions base on the data, and $x_{HO}$is the hypothesized handover position. $P(x_{HO}|C)$ represents the prior belief where a handover will take place given that the handover is natural. The prior $P(C)$ in Equation (1) is a factor which represents the trust of the receiver that a natural handover *(C=N)* or an unnatural handover *(C=UN)* will occur. Since in cooperative tasks the receiver does not expect to be surprised by an unexpected act performed by his partner, we assume that the expectation of having a natural handover *P(C=N)* is bigger than of having an unnatural handover *P(C=UN). P(C=N)* and *P(C=UN)* must add to 1:

$P(C=N)+P(C=UN)=1$ (2)

The normalization by the denominator $P(s)$ in Bayes’ theorem must be chosen such that the sum of $P(C=N|x_{HO},s)$ and $P(C=UN|x_{HO},s)$is equal to 1. We thus obtain for the normalization factor:

$P(s)=P(s|C=N,x_{HO})P(x_{HO}|C=N)P(C=N)+P(s|C=UN,x_{HO})P(x_{HO}|C=UN)P(C=UN)$ (3)

To infer if there will be a natural or an unnatural handover can now be reformulated as a combination of $P(s|C,x_{HO})$ and $P(x_{HO}|C)$, which are the endpoint estimation and the handover position prior distributions. In the following we will denote $P(s|x_{HO})P(x_{HO}|C)$ as the likelihood. In our model we assume the endpoint estimate to be independent of the handover case, therefore the handover case *C=N/UN* can be dropped:

$P(s|C=N,x_{HO})=P(s|C=UN,x_{HO})=P(s|x_{HO})$ (4)

For the unnatural case the handover position prior $P(x_{HO}|C=UN)$ is neglected and therefore is set to a uniform distribution. According to the model assumptions Equation 1 becomes to:

$\begin{matrix} P(C=N|x_{HO},s)=\frac{P(s|x_{HO})P(x_{HO}|C=N)P(C=N)}{P(s|x_{HO})P(x_{HO}|C=N)P(C=N)+P(s|x_{HO})(1-P(C=N))} \\ P(C=UN|x_{HO},s)=\frac{P(s|x_{HO})(1-P(C=N))}{P(s|x_{HO})P(x_{HO}|C=N)P(C=N)+P(s|x_{HO})(1-P(C=N))} \end{matrix}$ (5)

Furthermore, we assume all distributions to be Gaussian. Therefore, endpoint estimation and prior about the handover position have the uncertainties $\Sigma_{s}$ and $\Sigma_{HO}$ correspondingly. Because both are Gaussians, the likelihood for the natural handover case is also a multivariate Gaussian with a new expectation value $\mu_{N}$ and covariance matrix $\Sigma_{N}$:

$P(s|x_{HO})P(x_{HO}|C=N)=\frac{1}{\left(2\pi\right)^{3/2}\left|\Sigma_{N}\right|^{1/2}}\exp\left[-\frac{1}{2}\left(s-\mu_{N}\right)^{T}\Sigma_{N}^{-1}\left(s-\mu_{N}\right)\right]$ (6)

where $\mu_{N}$ and $\Sigma_{N}$ are calculated by:

$$\begin{matrix} \mu_{N}=\left(x_{HO}^{T}\Sigma_{HO}^{-1}+s^{T}\Sigma_{s}^{-1}\right)\left(\Sigma_{HO}^{-1}+\Sigma_{s}^{-1}\right)^{-1} \\ \Sigma_{N}=\left(\Sigma_{HO}^{-1}+\Sigma_{s}^{-1}\right)^{-1} \end{matrix}$$

For a handover to an unnatural position the handover position prior is set to be uniformly distributed. Thus, the likelihood for the unnatural handover case can be written as:

$P(s|x_{HO})=\frac{1}{\left(2\pi\right)^{3/2}\left|\Sigma_{UN}\right|^{1/2}}\exp\left[-\frac{1}{2}\left(s-\mu_{UN}\right)^{T}\Sigma_{UN}^{-1}\left(s-\mu_{UN}\right)\right]$ (7)

where $\mu_{UN}$ and $\Sigma_{UN}$ are calculated by:

$$\begin{matrix} \mu_{UN}=s \\ \Sigma_{UN}=\Sigma_{s} \end{matrix}$$

In other words, the handover case (*N* or *UN*) depends on whether or not the handover position prior improves the prediction of the endpoint estimation.

Because $P(N|x_{HO},s)$ and $P(UN|x_{HO},s)$ add to 1, our model reports a natural handover when the calculated posterior probability is higher than $P\left(N|x_{HO},s\right)>\frac{1}{2}$.

**Model implementation:**

The model was implemented and simulated in Matlab. To reduce the complexity of the model, the simulation was implemented in two planar dimensions. For the experiments 1 to 3 we generated minimum jerk trajectories with endpoints corresponding to the experimental condition superimposed with Gaussian noise in longitudinal and transversal directions. For the robot experiments 4b and 5b, the two-dimensional projections of the robot trajectories used in the experiments were fed into the model.

The model simulated the observation, estimation, and decision process over time (model overview see Fig. 5) using a temporal resolution of 100 time steps for the duration of the delivering movement. At each time step, the model received a new partial observation of the trajectory and first estimated the endpoint and the reliability of the partially observed trajectory. Then the posterior probabilities for unnatural and natural handover positions were calculated and compared. Finally, the covariance matrix of the posterior probability distribution of the endpoint estimate was used to decide if the reliability of the predicted endpoint was sufficient to start the receiver’s movement, resulting in the simulated reaction times reported in Fig. 8.

**Endpoint estimation and prior**

This module used the observed part of the trajectory to estimate the direction and length of the delivering movement. The values for direction and length were calculated separately. The length of the movement was estimated using nonlinear regression (Matlab function nlinfit) by fitting a minimum jerk trajectory to the observed noisy trajectory. At the beginning of the observation only a very short part of the trajectory is available. In this case the nonlinear regression often failed to converge and stopped due to an iteration limit. If that happened, we randomly assigned a point on the table as endpoint. The direction of the endpoint was estimated by calculating the mean direction of the given part of the trajectory.

The observation-time dependent covariance matrix Σ*_s_(t)* for the endpoint-estimation probability density function was calculated using a Monte-Carlo simulation. For each observation step 1000 noisy minimum jerk trajectories were generated and fed into the endpoint estimation. The covariance matrix was then calculated from the distribution of the 1000 endpoints. Fig. S1 shows the development of the diagonal elements of observation-time dependent covariance matrix Σ*_s_(t).* The covariance matrix for the natural prior Σ*_HO_*= [0.035^2^ 0; 0 0.055^2^] was calculated from handover position distributions collected during previously performed experiments [13].

**Figure S1: SD of the distance and direction of the endpoint estimates derived from movement observation over time**
The figure shows the development of the diagonal elements of observation-time dependent covariance matrix Σ*s(t)* for the endpoint-estimation, expressed in SD. Note that directional variability is also expressed in meters. It can be seen that the direction estimate is converging faster than that of the overall distance of the movement. Since the diagonal elements of the covariance matrix remain close to zero, the decision variable is basically a combination of these two variabilities.

**Simulation parameters**

We synthesized minimum jerk trajectories to different handover positions in accordance with experiment 1-3. From each trajectory, 1000 data points were sampled. Each duration was normalized to 1 sec. The length of the straightforward movement towards the natural handover position was 0.3 m. In longitudinal and transversal direction a Gaussian noise was added with σ = 0.025 m. For displaced handovers (experiments 2 and 3, 0.2 m to the left or right) the straightforward trajectory was rotated and stretched to match the desired final handover position. Thus, all simulated trajectories matched the average experimental trajectory in start point and duration. For each time step of the simulation the length of the observed trajectory was increased in proportion of the elapsed time. The decision whether the reliability of the estimated handover position was sufficient to react was made by calculating the mean variance of the posterior distribution. If this value fell below a decision threshold ε = 0.003, the simulation was stopped and the respective time was taken as reaction time.

For all simulations we chose the probability of a handover at a natural position *P(C=N)* = 0.75, which means that the receiver assumes no surprise during the experiment.

The learning rates for the handover position prior’s mean and covariance matrix were chosen to be $\beta_{\mu}=0.9$ and $\beta_{\Sigma}=0.1$correspondingly.

**Robot experiments adjustments**

For the robot human experiments the trajectories were normalized to a duration of 1.2 seconds as used experimentally. In none of the experiments with the robotic partner a significant decrease of the reaction times over trials was found. To prevent a decrease of the reaction times in the model, the covariance matrix for the prior must not change. Thus, for the simulations of the robot experiment the learning rates for the prior of the handover position were set to$\beta_{\Sigma\mu}=0$.

The overall differences in the reaction times between the experiments performed with a humanoid and an industrial robot were simulated by adjusting the decision value threshold. This can be interpreted as following: if the deliverer has not a human-like appearance or human joint configuration the receiver needs to predict the partner’s actions with a higher degree of confidence. Thus, in experiment 5 where the industrial robot delivered the cubes, we divided the threshold by the factor 2 resulting in ε = 0.0015. No other parameter was changed between human and robot experiments.

**Figure S2: Trajectory and velocity profile of a movement with evidence for online correction.** Left: Trajectory shown from above. R and M denote the right and middle position for delivering the cube (unknown to the test subject). Right: Velocity profile of the movement.

**Online correction example:**

Figure S2 shows an example of one of the 7 trajectories with evidence for online correction during the receiver’s movement. The cube was passed to position R (20 cm to the right of the deliverer). At the beginning the direction of the movement was pointing towards the middle position M. Later the movement was corrected towards position R. In the velocity profile such a correction is visible as valley.
